# High Resolution On-Road Air Pollution Using a Large Taxi-Based Mobile Sensor Network

**DOI:** 10.3390/s22166005

**Published:** 2022-08-11

**Authors:** Yuxi Sun, Peter Brimblecombe, Peng Wei, Yusen Duan, Jun Pan, Qizhen Liu, Qingyan Fu, Zhiguang Peng, Shuhong Xu, Ying Wang, Zhi Ning

**Affiliations:** 1 Division of Environment and Sustainability, The Hong Kong University of Science and Technology, Hong Kong SAR, China; 2 Department of Marine Environment and Engineering, National Sun Yat-sen University, Kaohsiung 804201, Taiwan; 3 Shanghai Environmental Monitoring Center, Shanghai 200030, China; 4 Shanghai Eureka Environmental Protection Hi-Tech Ltd., Shanghai 200090, China; 5 Sapiens Environmental Technology Co., Ltd., Hong Kong SAR, China

**Keywords:** Shanghai, motorways, roads, mobile network, COVID-19, NO_2_, PM_2.5_, CO

## Abstract

Traffic-related air pollution (TRAP) was monitored using a mobile sensor network on 125 urban taxis in Shanghai (November 2019/December 2020), which provide real-time patterns of air pollution at high spatial resolution. Each device determined concentrations of carbon monoxide (CO), nitrogen dioxide (NO_2_), and PM_2.5_, which characterised spatial and temporal patterns of on-road pollutants. A total of 80% road coverage (motorways, trunk, primary, and secondary roads) required 80–100 taxis, but only 25 on trunk roads. Higher CO concentrations were observed in the urban centre, NO_2_ higher in motorway concentrations, and PM_2.5_ lower in the west away from the city centre. During the COVID-19 lockdown, concentrations of CO, NO_2_, and PM_2.5_ in Shanghai decreased by 32, 31 and 41%, compared with the previous period. Local contribution related to traffic emissions changed slightly before and after COVID-19 restrictions, while changing background contributions relate to seasonal variation. Mobile networks are a real-time tool for air quality monitoring, with high spatial resolution (~200 m) and robust against the loss of individual devices.

## 1. Introduction

In recent decades, road transport has been an increasing source of air pollution, especially in Chinese cities. Road transport adds to a range of pollution sources that arise through the demands of urban infrastructure and energy consumption, so pollutant emissions and the resultant air pollution is a common phenomenon in urban areas [1,2,3,4]. Traffic-related air pollution (TRAP) makes a vast contribution to the total air pollution of most cities, such that traffic emissions are of particular concern in urban areas [5,6,7]. Road transport pollutants represent a major source of carbon monoxide (CO), nitrogen dioxide (NO_2_), and fine particulate matter (PM_2.5_, *d*_p_ < 2.5 μm). More than 50% of the world’s population lives in urban areas, and many are currently exposed to air pollution levels that exceed WHO limits [8,9]. This makes urban air pollution a major environmental and social problem [7,10,11,12,13], especially in megacities [14,15]. Exposure to elevated air pollution concentrations also gives rise to adverse effects on public health [16,17]. Hence, it is important to measure the on-road pollutants and effectively control them.

Among the three major urban agglomerations in China, the Yangtze River Delta Urban Agglomeration has the largest population, the greatest area, and the highest GDP [18,19]. However, air pollution associated with rapid economic growth presents an obstacle to sustainable development. Shanghai, with over four million registered vehicles, is one of four municipalities administered directly by the central government of China, so traffic emissions have attracted significant attention [20].

Since 2012, 19 air quality monitoring stations (AQMS) have been set up in different districts of Shanghai for regulatory purposes to monitor the long-term trend of criteria pollutant concentration in the city. However, the high installation and maintenance costs of AQMS greatly limit the number of stations and the spatial coverage. Their representativeness of the heterogeneous air pollution distribution in the city is often challenged by different land use types and varying pollution concentrations driven by the physical and chemical transformations of roadway emissions from on-road sources to roadside and ambient [21,22,23,24]. Different studies have attempted to characterise fine scale urban air pollution distribution, using satellite data inversion, land use regression, or regional/micro-scale air quality models to interpolate air pollution data from the AQMS network.

However, a knowledge gap remains regarding the dynamic characteristics of traffic emissions from roadway networks and their contribution to fine scale air quality [25]. In the past decade, the mobile monitoring approach as an efficient tool to measure on-road air pollution with high spatial resolution has become increasingly important [26,27,28]. It represents a valuable tool for policymakers and environmental protection agencies, providing spatially resolved and real-time patterns of air pollution that are robust against the loss of individual sensors. They may reveal the intensity and persistence of places with high concentrations. The recent development of air sensors has significantly increased the temporal and spatial resolution of such monitoring with real time positioning using mobile platforms for a variety of applications. Wei et al. [29] deployed mobile sensors on 14 buses in Hong Kong with over 5000 trips covering the major roadway sections and developed methods to distinguish background and local air pollution. Apte et al. [30] used two Google Street View vehicles in a 30 km^2^ area of Oakland, CA to measure NO, NO_2_, and black carbon at the 30 m scale over one year and characterised the localised pollution hotspots to address air quality data gaps. Yu et al. [31] used taxi-based and fixed site monitors to examine the spatial distribution particulate matter in Jinan; the study covered an area of 10,200 km^2^ for over a month as a demonstration of the methodology. Earlier studies have often been limited by the range of pollutants, number of mobile platforms deployed, the duration of the monitoring campaign, or a spatial coverage insufficient to represent the roadway network.

This study is the first to monitor the variation of traffic-related air pollution using a mobile sensor network. A total of 125 urban taxis in Shanghai were equipped with a monitoring device to determine the concentrations of carbon monoxide (CO), nitrogen dioxide (NO_2_), and PM_2.5_ from November 2019 to December 2020, which allowed the spatial and temporal patterns of pollutants on the road to be characterised. The research aims to: (i) demonstrate the effectiveness of the quality control and quality assurance protocol for large scale mobile monitoring; (ii) investigate the impact of the deployment strategy on roadway coverage and number of mobile devices required; and (iii) characterise the temporal and spatial profiles of on-road pollution concentrations at different times of day and year and on different roadway types; (iv) apportion the contribution of urban background and localised traffic contributions to on-road pollutant concentrations.

During the monitoring campaign, the mobile sensor network collected over 15 million data points at 5 s resolution and the fleet travelled a total of 27,512,738 km. The pollution concentration and corresponding position data were further assimilated and assigned to 51,118 pre-defined segments with 200 m unit length. Clear spatial patterns and distinct temporal profiles of the pollution distribution emerged from diurnal traffic flow, commuting between the city centre and peripheral areas, and unevenly distributed pollutants from the vehicle fleet. The impact of COVID-19 lockdown on traffic pollutant concentration levels and distribution was also observed demonstrating the sensitive response of the mobile sensor network to changes in traffic patterns.

This study increases the scale and duration in comparison with earlier work. The findings from this work serve as an important reference for designing such mobile networks in cities and the related operational protocols.

## 2. Materials and Methods

### 2.1. Roadway Type Classification

This study was conducted in Shanghai, which has an area of 6341 square kilometres and a population of nearly 28 million. The mobile air monitoring campaign began in December 2019 following one month of trial operation in November 2019.

**Figure 1:**
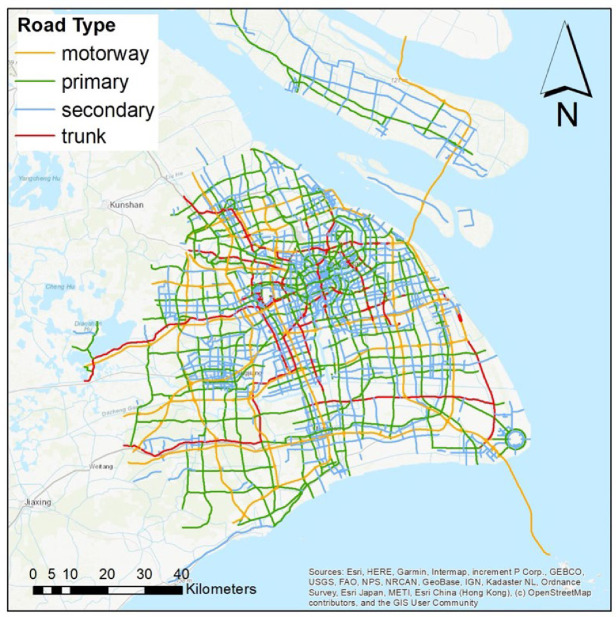
Map of major study roads in Shanghai—Map credit: OpenStreetMap.

The roadway classification was based on *OpenStreetMap* (OSM), an open-source geographic information database with comprehensive roadway coverage and classification [32,33]. Studies have shown that OSM data for prefecture-level cities in China are almost complete [33], and the positioning accuracy is reportedly high [32,34,35]. OSM has predefined 23 road types, including trunk, primary, secondary, and tertiary roads, along with motorways, bike paths etc. This study has adopted OSM definitions to facilitate data analysis and includes four road types to balance coverage and complexity: motorways (limited-access highways for long-distance, heavy, or fast traffic), trunk roads (the most important roads in a system after motorways; largely pre-existing roads), primary roads (important roads that often link towns or main roads within cities), and secondary roads (not part of a major route, but nevertheless forming a link in the national route network). Figure 1 shows the coverage by road types and they cover approximately 57% (7432 km) of the total road length in Shanghai (~13,000 km in 2019). The selected road types have varying width with double and multiple lanes. To simplify the data analysis, we set 10 m to represent the roadway width in each direction. This provides a sufficient buffer to include the position-tagged pollutant concentration data from the mobile sensor network for analysis.

The mobile sensor used here reports the average pollution concentration every 5 s, typically spanning a travelling distance of 28–167 m with average traffic speed ranging between 20–120 km h^−1^, the same speed range found in this study. We set 200 m as the unit length of roadway segment for analysis to accommodate the different traffic conditions and the mobile monitoring data at a specific time is assigned to the pre-defined segments as input for spatial data analysis. Finally, the four road types were divided into a total of 51,118 segments, each with a buffer of 200 × 20 m covering two directional traffic.

ArcMap (Version 10.8.1, Esri, Redlands, CA, USA) was used to split the road polygons into segments, and the data was exported to Rstudio (Version 1.4.1103) afterwards for further analysis, such as GPS point positioning and pollutant concentration calculation.

### 2.2. Description of the Mobile Sensor Network

**Figure 2:**
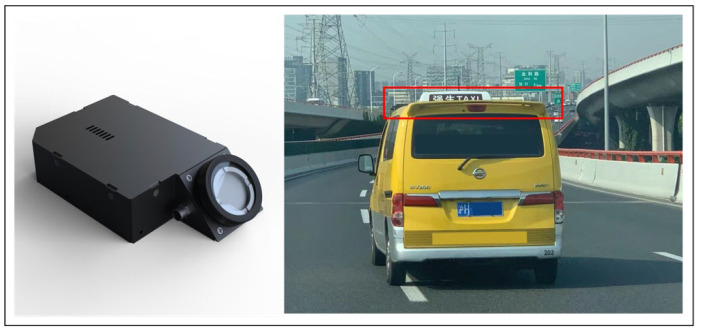
The mobile devices installed on the rear fins of a taxi.

We deployed 125 mobile air monitoring devices on the fleet of electric taxis in Shanghai. The mobile monitoring devices are lightweight and modular (weight: ~2 kg; size: 177 mm × 109.5 mm × 48.3 mm) with active flow design. They were housed in the enclosure at the rear top of the taxis with air inlet reaching out and pointing towards the driving direction (Figure 2).

The data telemetry has low power consumption (5 V and <3 W) and runs on external battery modules or solar power enabling the mobile devices to be suitable for real-time monitoring of particulate matter and different gases under mobile conditions. Several functional components are included (Table 1): (i) The dynamic baseline tracking electrochemical sensor module (Model PDF-4, Sapiens, Hong Kong, China) is used to measure CO and NO_2_ concentrations. The module contains an original sensor head from a Type A 4-electrode electrochemical sensor from Alphasense (CO-A4 and NO_2_-A43F) and a corresponding proprietary baseline sensor head to provide the original signal. The concentration range limit for CO is 20 ppm and 5 ppm for NO_2_, and the specifications for response time and operating life are shown in Table 1. Each gas module is based on a proprietary dynamic reference tracking technology electrochemical sensing and Pair Differential Filtering (PDF) technology to eliminate ambient temperature and humidity effects on the measurements [36,37,38]. The gas sensor module was embedded and sealed in a non-reactive polytetrafluoroethylene (PTFE) manifold. (ii) A laser-scattering-based sensor PMSX-003 (Plantower, Beijing, China) was used to measure PM2.5 concentration. It can continuously count the number of suspended particles of different sizes and convert it to mass concentration [12]. (iii) A temperature (T) and relative humidity (RH) sensor was attached to the gas sample tube to measure the meteorological conditions of the air entering the equipment. (iv) A GPS module that can receive satellite signals in 72 channels with low power consumption and high sensitivity was used. It quickly and accurately locates weak signals in cities, canyons, under viaducts, and inside cars. (v) A data transmission module outputs the pollutant concentration and device status data directly to the cloud server in real-time through the global system for mobile communications for online data processing. The mobile device maintains a sampling flow rate of 0.6 L min-1 and is equipped with a built-in micro diaphragm pump. The gas sampling adopts a pump suction active air intake, which can prolong the service life. A PTFE inline filter is mounted in the gas inlet and used to remove aerosols from the air and prevent damage to the gas sensor.

### 2.3. Data Analysis

Data collected using mobile monitoring devices include real-time CO, NO_2_, and PM_2.5_ concentrations, time, location (latitude and longitude), device ID, temperature, and relative humidity of each device in 5 s resolution (Table S1).

Despite the skewed distribution of the pollution concentration data, parametric statistical tests were sometimes used in this study because the number of observations was large (~1.5 million points for each month), so the central limit would allow parametric methods, such as Welch’s *t*-test and ANOVA, to be valid. Means and standard deviation represent most data, but occasionally where the data had a large span, medians and quartiles were used in box whisker plots, with the box defining the lower (*Q*_1_) and upper quartile (*Q*_3_) and the whiskers of 1.5 times the interquartile range.

Self-contamination was estimated from the concentration distributions of each pollutant at different speeds and is probably small, as the concentrations at low driving speed (<5 km h^−1^) were not significantly higher than those measured at other speeds.

### 2.4. Sensor Network Quality Assurance and Quality Control Protocol

Mobile air monitoring devices are often criticised for deficiencies and limitations in data accuracy [39,40,41], so we employed a set of strict quality assurance and quality control (QAQC) protocols. These included (i) pre-deployment calibration, (ii) regular examination and maintenance after deployment, and (iii) data monitoring and error detection. Quality assurance (QA) typically minimises data inaccuracies through regular inspections and maintenance, continuous monitoring, and evaluation of the behaviour of devices in the field, which can prevent measurement failures or detect problems earlier. Quality control (QC) is performed during the monitoring through calibration after deployment and involves automatic or semi-automatic cross-checking of values against predetermined criteria, such as trend analysis for sensor drift detection [42,43].

Pre-deployment calibration of the sensitivity and the baseline of each sensor was carried out using a Dynamic Gas Calibrator (Model 146i, Thermo Scientific, Waltham, MA, USA) and Zero Air Supply (Model 111, Thermo Scientific) to generate stepped concentrations of NO_2_ and CO in the laboratory. Calibration of PM_2.5_ was carried out through side-by-side comparison with a reference analyser (Thermo Scientific, Model 1405). Cross-sensitivity to different pollutants and response to ambient temperature and humidity was validated in the field [37,38]. After calibration, the mobile devices were placed next to the air quality monitoring station at Shanghai Normal University (31°09′55.4″ N 121°24′36.0″ E) for five days for verification before deployment.

The concentrations of CO, NO_2_, and PM_2.5_ measured by the devices were not significantly different from those of the standard instrument (α = 0.05, ANOVA). The coefficient of determination (*R*^2^) for each pollutant (CO, NO_2_, PM_2.5_) was generally high (CO: 0.99, NO_2_: 0.85–0.93, PM_2.5_: 0.88–0.91). These *R*^2^ values suggest measurements are well correlated compared to previous studies [44]. The results show that the mobile devices compare reasonably well to reference analysers. The *R*^2^ for the linear regression is between 0.85 and 0.99, which suggests the data have robust quality under varying ambient conditions. The normalised mean square error (NMSE) and the correlation coefficient have been used as recommended by Hanna et al. [5,45] and applied by Wu et al. [46] for PM_2.5_ concentrations. The recommended criteria interval for the correlation coefficient is larger than 0.8, and the best agreement is 1 for NMSE below 0.5 and 0 [47]. The equation, recommended criteria, and best agreement for all of the methods are shown in Table 2. The measurements are all within the recommended criteria interval, which is also very close to the best agreement, indicating consistency between the values from the two sources. Thus, the data collected by the mobile devices can provide a reliable research basis for the evaluation of urban air quality.

Regular examination and maintenance were also conducted during the deployment. The mobile devices were called back batch-by-batch every two weeks during the first three months of the monitoring campaign to assess the effectiveness of the operation protocol and refine the frequency of maintenance. The relative deviation of the measured standard gas concentration at the time of device recall was less than 15%, over periods ranging from one month to two months.

We monitored data quality through real-time cloud-based data streams daily. By comparing it with the surrounding AQMS, the outliers, instances of malfunction, and baseline and sensitivity drift can be detected and resolved to ensure data quality. Since the device is lightweight and low-cost, there are some trade-offs in data quality. Data were deleted based on two criteria: (i) data points on unselected road types, such as tertiary roads, (ii) abnormal or invalid data after QAQC. Combined with road segmentation, we integrated the data into hourly averages for each segment point for ease of analysis.

### 2.5. Estimation of Local Traffic-Based Contribution to On-Road Pollutant Concentration

On-road pollutant concentration is attributed to two components: one is the background signal due to the aggregated urban pollution impact but not directly related to local traffic emissions, while the other is the localised traffic contribution to the elevated pollutant concentrations. The two signals have different profiles and frequency of variations. Low percentiles can represent the background concentrations, i.e., not affected by peak signals and exhibiting only slow variation [29,48,49]. We took this approach and used the 5th percentile to estimate background signals:

*C*_BG,*i*,*t*_ = *C*_T, 5%percentile_ (1)

*C*_LC,*i*,*t*_ = *C*_T,*i*,t_ − *C*_BG,*i*,t_ (2)

*P*_LC,*i*,*t*_ = *C*_LC,*i*,*t*_/*C*_T,*i*,*t*_ (3)

where *C*_BG,*i*,*t*_ represents the background air pollutant concentration for the pollutant *i* and time *t* (we used the 5th percentile concentrations during hour *t*). The parameter *C*_LC,*i,t*_ represents the local emission concentration for pollutant *i* at time *t* and was calculated by subtracting *C*_BG,i*,t*_ from the raw measurement *C*_T,*i,t*_ (measured concentration for the *i*th pollutant at time *t*) minus *C*_BG,*i,t*_. The local pollutant contribution was calculated from the ratio of *C*_LC,*i,t*_ and *C*_T,*i,t*_. Preliminary analysis showed the choice of the 5th percentile was reasonable as there was a good agreement (Mann–Whitney test NO_2_: *W* = 156,917, *p* < 0.0001; CO: *W* = 536,556, *p* < 0.0001, PM_2.5_: *W* = 269,835, *p* < 0.0001 and *t*-test: NO_2_: *t* = −17.041, *df* = 1039.5, *p* < 0.0001; CO: *t* = 65.445, *df* = 733, *p* < 0.0001, PM_2.5_: *t* = 2.7654, *df* = 1274.5, *p* = 0.0058) between the average hourly concentrations of PM_2.5_ and CO from the 10 stations of Shanghai AQMS network and the 5th percentile concentration measured by the taxis (see Supplementary Figures S1 and S2). The agreement was not as good for NO_2_, when on-road concentrations were high.

## 3. Results

### 3.1. Speed Distribution and Roadway Coverage

During the data collection period, about 300,000 raw data points were obtained per day. After alignment of the road segments and data assimilation, more than 160,000 data points remained and were assigned to the 51,118 road segments across all road types, so the network collected from 0 to 460 segments of data per day. Still, the vast majority of road segments have only one or two data points (average 4.9, median 1.3, and *Q*_1_ = 0.3 and *Q*_3_ = 4.7). Primary roads account for the highest proportion of segments with 15,975 segments (31.3%), followed by secondary roads (15,508; 30.3%), motorways (12,800; 25%), and trunk roads (6835; 13.4%). The average speed of vehicles on all streets is 45.1 km h^−1^, ranging from 1.0 km h^−1^ to 122.0 km h^−1^, with a 43.7 km h^−1^ median; and *Q*_1_ = 28.4 km h^−1^ and *Q*_3_ = 58.8 km h^−1^ (which includes all the data when the taxi is operating).

**Figure 3:**
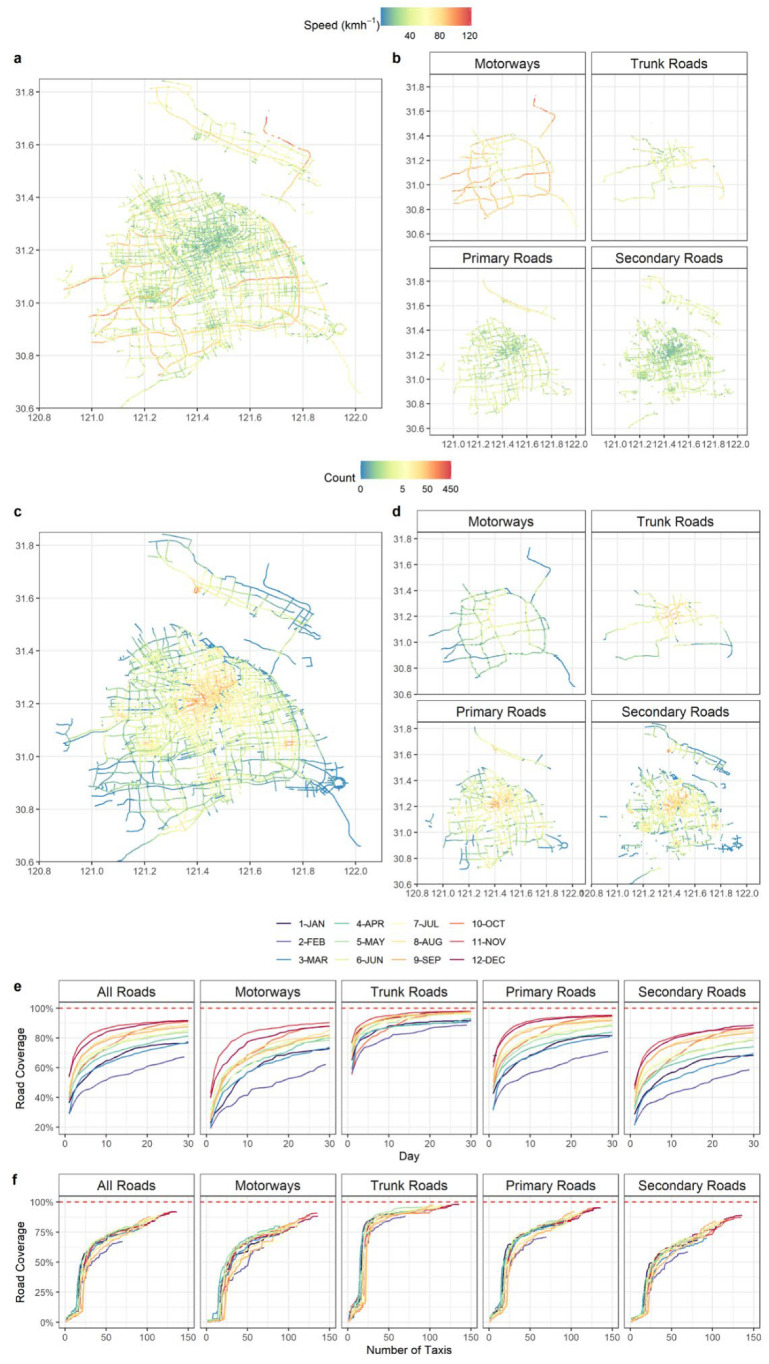
Spatial distribution of the speed (**a**,**b**) and number of points in each segment (**c**,**d**) on different types of roads in Shanghai, and the day and cumulative number of taxis over 12 months (**e**,**f**).

Figure 3 shows the spatial distribution of the speed and the number of data points on different types of roads in Shanghai. The average speed of vehicles travelling in the city centre is generally below 40 km h^−1^, while it increases to 60 km h^−1^ on roadways on the periphery of the city following a typical urban traffic profile. The cross comparison of different roadway types shows the highest vehicle speeds occur on the motorway, up to 120 km h^−1^, while the speed becomes lower in secondary roads, typically below 40 km h^−1^. The variation in vehicle driving speeds results in the non-uniform assignment of data points on road segments, as shown in Figure 3c,d. The density of data points is highest in the city centre (red), showing more frequent taxi trips, and lower in other parts of the city (blue). Figure 3e presents the relationship between the percentage of roadway coverage and the number of monitoring days in different months. Trunk roads are the most frequently travelled road type, showing the highest coverage rate compared with others. The fleet of 125 taxis was able to cover 80 to 95% of the trunk roads in 10 days. On the other hand, roads have the lowest coverage rate, probably due to the greater total length and fewer trips by taxi. For all road types, the months of January, February, and March had significantly lower coverage than the rest of the year, a clear reflection of the impact of COVID-19 on the reduction in overall roadway traffic. In addition, Figure 3f presents the monthly road coverage rate with the increasing number of taxis in the fleet. There is no noticeable difference in road coverage in different months, and in general 80 taxis could achieve 80% road coverage. There are slight differences between road types, for example, trunk roads only need 25 taxis for 80% coverage in a month, while secondary roads need at least 100 taxis to achieve the same coverage.

Statistical data for the various roads is summarised in Table 3. Trunk roads have the smallest number of segments and the shortest length, yet the highest average monthly coverage of more than 95%. On motorways, the average driving speed of vehicles can reach 64.6 ± 20.2 km h^−1^, so the number of points contained in each segment is the smallest. Primary roads have up to 15,975 segments, and an average monthly coverage rate of 88%. Secondary roads have the longest total length (2563 km) and the largest number of data points in each segment. The slowest driving speed is found on secondary roads, with an average of 33.9 ± 15.1 km h^−1^. The average monthly coverage for all types of roads is 84%, and the average driving speed is 45.1 ± 20.9 km h^−1^.

The unique scale and long-term monitoring of the mobile network in this study shows that the deployment strategy and study design are very relevant to urban traffic characteristics, but the relationship between the number of mobile devices and road coverage rate is not linearly correlated. The findings may provide a useful reference in the design of mobile monitoring studies. It should be noted the taxi-based mobile network is characterised by a random route and trip schedule, which is very different from a bus-based mobile network, in which the routes are fixed and often repeated with routine schedules [29].

### 3.2. Spatiotemporal Analysis

The monitoring campaign ran from 1 December 2019 to 21 December 2020, with the first month for a trial run and the following complete year 1 January 2020 to 21 December 2020 for the analysis. In the following sections, changes in pollutant concentrations will be examined: (i) spatial analysis for different kinds of road segments and (ii) diurnal analysis for hourly averages of pollutants during weekdays and weekends, and each month.

#### 3.2.1. Spatial Analysis of Air Pollutant Concentration

**Figure 4:**
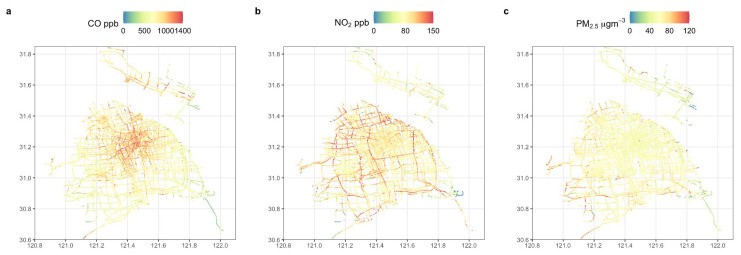
Spatial distribution of the average hourly concentrations of CO (ppb) (**a**), NO_2_ (ppb) (**b**), and PM_2.5_ (μg m^−3^) (**c**) in Shanghai from January 2020 to December 2020.

The annual average concentrations of CO (ppb), NO_2_ (ppb), and PM_2.5_ (μg m^−3^) on the corresponding road segments along the entire roadway network for 2020 are shown in Figure 4. The concentration of CO can reach ~1400 ppb in the city centre, while it is relatively low in rural areas (~30 ppb), indicating that there is a relationship between petrol vehicle activity and CO emissions. However, the concentration of NO_2_ is higher on most motorways, and the speed on these roads is relatively high, probably indicating that as vehicle speed increases, so do NO_2_ emissions. The PM_2.5_ concentration is relatively low in the west, as traffic activity is lower than in the city centre. However, there are likely to be important non-traffic sources in addition to secondary sources, which may relate to different land use, and restrictions on vehicle types.

**Figure 5:**
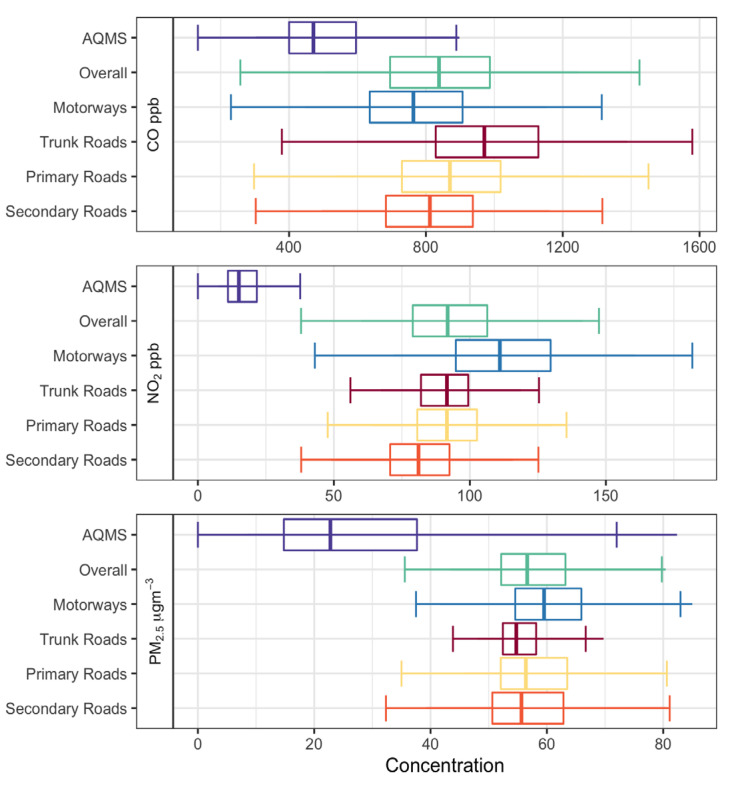
Boxplots for hourly average variation for CO (ppb), NO_2_ (ppb), and PM_2.5_ (μg m^−3^) in Shanghai from January 2020 to December 2020.

The hourly average concentrations of CO, NO_2_, and PM_2.5_ on the four road types are 854 ± 258 ppb, 95 ± 28.7 ppb, and 56.1 ± 7.7 μg m^−3^, respectively, as shown in Table S2. As can be seen from the boxplot in Figure 5, the highest CO concentration is 987 ± 275 ppb on the trunk roads, and the lowest is on the motorways (where speeds are higher; 65 ± 20.2 km h^−1^), which suggests that the CO level generally decreases with an increase in average speed. The on-road CO concentration shows a peak with change of speed, indicating a specific range of traffic flow. However, the number of vehicles is a stronger control on pollution levels than the speed of cars (Rashidi and Massoudi, 1980). Besides, the highest concentration of NO_2_ is 113 ± 28 ppb on motorways and the lowest concentration is on secondary roads (82 ± 23.4 ppb), indicating that increased velocity also increases the NO_2_ concentration. In addition, the PM_2.5_ concentration is also higher on the motorways with an average of 61.2 ± 14.4 μg m^−3^, which may be caused by frequent acceleration and deceleration of vehicles during stops and starts. These activities are known to increase tailpipe emissions, as well as brake lining and tire wear [50]. Sanders et al. [51] and Garg et al. [52] showed that brake wear PM_2.5_ emission rates for light duty vehicles increase as the deceleration rate increases. Therefore, we conclude that PM_2.5_ concentrations are largely independent of road type, with NO_2_ differing by about 30 ppb on motorways (highest) from secondary roads (lowest). CO varies by about 200 ppb on trunk roads (highest) from motorways (lowest). As the speed of the taxis increases on different road types, NO_2_ and PM_2.5_ concentrations increase in parallel. However, the variation of CO concentration is related not only to the speed of vehicles but also to the number of vehicles and other factors.

**Figure 6:**
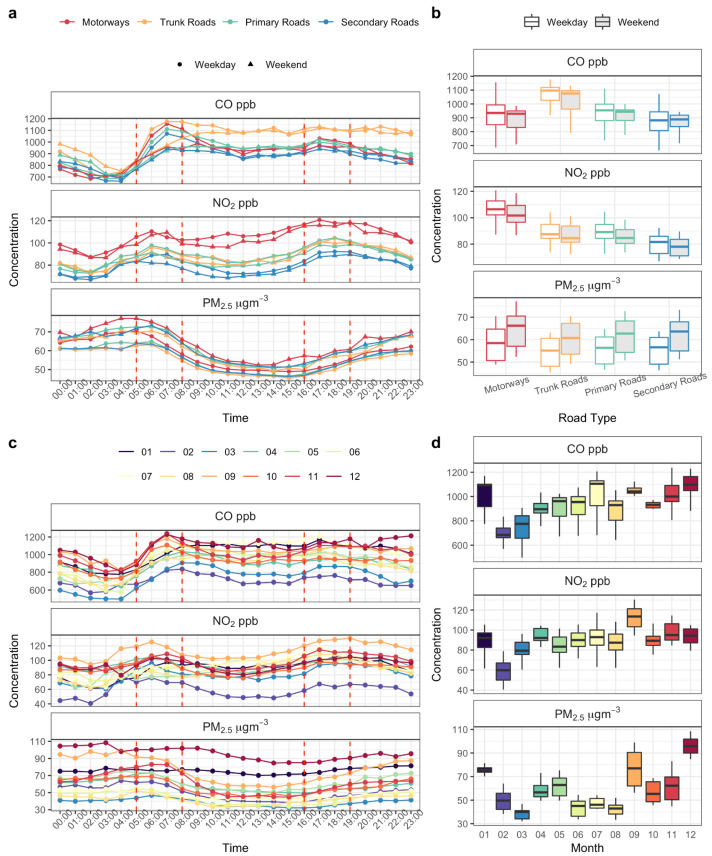
Daily cycles of the three pollutant concentrations measured by the mobile devices during peak/non-peak hours, weekdays/weekends, in each month in 2020. (**a**) Diurnal concentration change among different road types and between weekdays (dots) and weekends (triangles), with the dashed red line for peak hours from 05:00 to 08:00 and 16:00 to 19:00. (**b**) Statistics and overall distribution of four types of road, each box extending from the 25th to the 75th percentile, weekday (unshaded) and weekend (shaded). (**c**) Diurnal changes over each month; (**d**) the data statistics and overall distribution and each box extends from the 25th to the 75th percentile.

As can be seen from Figure 5, the AQMS concentration among the three pollutants are all lower (Welch’s *t*-test *p* < 0.0001) than those of on-road concentrations. As AQMS are mostly placed on the roofs of residential buildings and other places away from the street, they are not immediately affected by vehicle emissions. An ANOVA test reveals that not only are the measurements from the various road types different from the AQMS measurements, but they are also different from each other. The difference between all the individual road types is significant for all pollutants at the *p* < 0.01 level (using the Tukey test), even though PM_2.5_ is more homogeneously distributed. For this reason, the data is split into the different road types for the temporal analysis, shown in Figure 6.

#### 3.2.2. Temporal Analysis

Results in Figure 6a represent the average hourly diurnal concentration patterns for weekdays (Monday to Friday) and weekends (Saturday and Sunday) for each road type. The statistics and overall distribution are shown in Figure 6b. The colours represent different road types. As can be seen from Figure 6, the CO concentration on trunk roads (orange) is higher than other roads over a day and NO_2_ concentration is higher on motorways (red). The concentration of NO_2_ and CO on most weekdays is higher than that on weekends, but PM_2.5_ is in contrast about 6 μg m^−3^ lower throughout the day. The average concentration of NO_2_ is 82.4 ppb on weekdays and 79.3 ppb on weekends, that of CO is 861 ppb on weekdays and 849 ppb on weekends, while the average concentration of PM_2.5_ is 53.7 μg m^−3^ on weekdays and 59.7 μg m^−3^ on weekends. Except for PM_2.5_, the differences were not significant using Welch’s two sample *t-*test (i.e., PM_2.5_: *p* = 0.0037; CO: *p* = 0.584; NO_2_: *p* = 0.233).

The diurnal profile between the weekday and weekend over the entire campaign year has interesting differences. For CO, there is a distinct rush hour peak in the morning, and the weekday morning rush hour peaks are much more prominent compared with weekends. The observation is a clear reflection of urban commuting patterns, since CO is the major emission from petrol vehicles, which is still the largest vehicle fleet in China for daily commuting. The NO_2_ diurnal profiles are different from CO, especially on different types of roads, which is a reflection of the diesel-powered vehicle fleet. On weekdays and weekends, the concentration changes share similar profiles, but the overall concentration is less during weekdays, possibly due to the reduced volumes of diesel vehicles in urban areas. According to Hu et al. [53], PM_2.5_ concentrations from the cities in the Yangtze River Delta region varied less on weekdays than on weekends. As we can see from the boxplot, there was a difference (around 6 μg m^−3^) in the concentration during most of the day. Thus, there was no significant change in PM_2.5_ concentration between weekdays and weekends during the study period. In addition, PM_2.5_ concentrations on weekdays and weekends showed a similar diurnal trend.

The diurnal changes for three pollutants each month are shown in Figure 6c. The concentration changes in NO_2_ and CO are both related to pollutant emissions for that month. The concentrations in February and March were lower than those in other months, which is related to the lockdown period. Thus, the direct impact of traffic emissions on these two gases is evident. The PM_2.5_ concentration shows a seasonal profile: higher in winter and spring, and lower in summer and autumn. This arises because Shanghai is more susceptible to atmospheric pollutants from the north of China in spring and winter [54].

### 3.3. Traffic-Related Local Pollution Contribution

**Figure 7:**
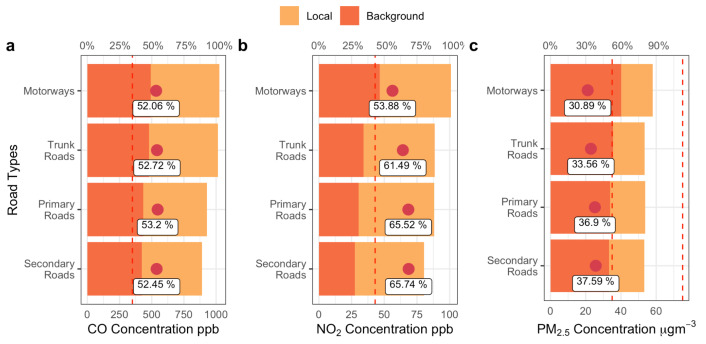
Local and background pollutant contributions to CO (**a**), NO_2_ (**b**), PM_2.5_ (**c**) for different road types (red for background contribution and orange for traffic-related emission contribution). The red dot is the contribution percentage for traffic-related local emissions. The dashed red line indicates the ambient air quality standard of China (after the unit conversion, the 24 h average limitation for NO_2_ is 43 ppb, CO is 350 ppb, 35 μg m^−3^ for the first level of PM_2.5_, and 70 μg m^−3^ for the second level of PM_2.5_).

Figure 7 shows the relative contribution of local and background concentrations of CO, NO_2_, and PM_2.5_ on different road types. Although the contribution varies among the pollutants, it is almost identical across road types (see boxed percentages in Figure 7). The locally derived contribution of CO varies the least (~1%) between different roads. This shows that the background contribution of CO on different roads is relatively constant, but CO exceeds the ambient air quality standard of China on all types of roads, so may imply a need for an overall reduction in emissions from traffic. The proportion of locally derived emissions to the concentration of NO_2_ shows the greatest variation among different types of roads, accounting for 66% on secondary roads and 54% on motorways, a difference of about 12%. Traffic speed on secondary roads is the lowest, and there are higher vehicle flows, so the locally derived pollution contribution on secondary roads is high. Except for motorways, where the background concentration slightly exceeds the ambient air quality standard of China, other road types generally have backgrounds that meet the standard. In contrast, PM_2.5_ shows the lowest variation of both concentration and locally derived contributions, displaying homogeneous distribution characteristics, a result of its wider dispersion and the role of secondary sources.

### 3.4. Concentration Comparison throughout the Entire COVID-19 Pandemic Period

Since the study spanned the outbreak of COVID-19, the variation of air pollutant concentrations across different response stages was investigated. Data (December 2019/July 2020) were divided into four stages: (i) December 2019/January 2020 before COVID-19, (ii) February 2020/March 2020 COVID-19 lockdown (iii) April 2020/May 2020 the recovery period in Shanghai (iv) June 2020/July 2020 the normal period post-COVID-19. Since the mobile devices measure concentrations on the urban road network, the road network carries a strong signal from variation in the traffic source. By contrast, national air quality monitoring stations are usually located away from major roads, reflecting city-wide variations.

**Figure 8:**
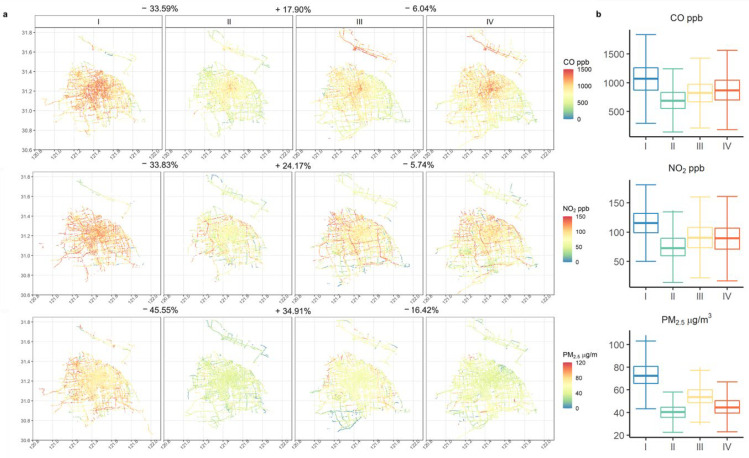
(**a**) Spatial changes of CO, NO_2_, and PM_2.5_ concentrations in four phases of the COVID-19 pandemic. (**b**) Boxplot for pollutant concentration distribution during each period of the COVID-19 pandemic.

Figure 8 shows the changes in the concentration of air pollutants (CO, NO_2_, PM_2.5_) during the four stages of the COVID-19 pandemic. We found that in the second stage the concentration of contaminants was the lowest: CO was reduced by 32% from the first stage, NO_2_ decreased by 31%, and PM_2.5_ dropped by 41%. The reductions are typical of changes experienced worldwide [55,56] and, in line with that of Wu et al. [57], are established from Shanghai’s fourteen air quality monitoring stations. It can be seen from the changes in spatial concentrations that CO is concentrated in the city centre for the first stage in high pollution areas, NO_2_ is found across the entire city, and PM_2.5_ is at the periphery of the city. High concentrations may be due to the significantly enhanced effect of northerly air flow in winter [58]. In the second phase of the epidemic (lockdown), the map shows that the concentration of pollutants drops dramatically. Very few streets are represented by red, and the PM_2.5_ concentration on some roads is <20 μg m^−3^. The areas with a high concentration of NO_2_ are restricted to a few motorways, and the locations with high CO concentrations are within a small distance of the city centre. When urban traffic was recovering after the lockdown, the three pollutants increased significantly (CO 38%, NO_2_ 25% and PM_2.5_ 37%); the change of NO_2_ on motorways is especially notable. In addition, the distribution of pollutants became more prominent, especially for NO_2_ on the motorways, but PM_2.5_ revealed a 22% decrease. This change may be due to the seasonal effect [59], which results in the highest concentrations of PM_2.5_ in Shanghai in November, December, and January, and lower concentrations in July, August, September, and October [60]. Therefore, from the third stage (April/May) to the fourth stage (June/July), the PM_2.5_ concentration drops noticeably.

## 4. Discussion and Conclusions

This study uses mobile air monitoring devices mounted on a large fleet of taxis (~125) to measure the air pollution on Shanghai roads. Quality control and quality assurance protocols ensured data were successfully collected by the mobile platforms and comparable to the data of the AQMS, thus ensuring accurate and high-resolution data. The integration of air sensors into mobile platforms offered a chance to evaluate the pollutants and study the spatial characteristics of the urban road environment, examining Shanghai motorways, trunk roads, and primary and secondary roads in 2020.

The results show that among the selected road types, trunk roads have the highest road coverage by the taxi fleet, while secondary roads have the lowest coverage, but they have the greatest length. The number of taxis running in each month varies: generally speaking, it takes 20 days for the average number of taxis on all roads in a month to reach 70% coverage, and it takes 50 taxis per month to reach 70% coverage on an annual basis. The measured CO concentrations are highest in the city centre, NO_2_ is most concentrated on motorways, while PM_2.5_ is higher away from the city. The concentrations of NO_2_ and PM_2.5_ increase with road speed, so predominate on motorways. All three pollutants have high concentrations from 05:00 to 08:00, while CO and NO_2_ are also high from 16:00 to 19:00. The concentrations of CO and NO_2_ are higher on weekdays than on weekends, while the concentration of PM_2.5_ is higher at weekends. Carbon monoxide has the highest concentration on trunk roads, while NO_2_ is highest on motorways; however, PM_2.5_ is rather more evenly distributed. Seasonally, NO_2_ concentration is lowest in winter, while PM_2.5_ concentrations are higher in spring and winter than in summer and fall; sometimes concentrations are twice as high. The concentration of each of the three pollutants decreased ~30–40% during the COVID-19 lockdown compared to the period before, thus this study provides insights into decreases in urban air quality during the COVID-19 pandemic period. However, surprisingly high levels of NO_2_ are apparent on motorways even under lockdown, suggesting the continuing importance of these roads.

Real-time air monitoring devices can be a valuable tool for policymakers and environmental protection agencies because they provide spatially resolved patterns of air pollution in real-time and are robust against loss of individual devices. They can potentially reveal the intensity and persistence of hot spots, though they create challenges because of the size of the dataset and the importance of integration with fixed monitoring sites.

## Figures and Tables

**Table 1:** Specifications of modules inside the mobile device.

Modules	Technique	Manufacturer	Technical Specification		
			Response Time, T_50_	Concentration Range Limitation	Linearity
CO module	Dynamic baseline tracking electrochemical sensors	Model PDF-4, Sapiens, Hong Kong, China	T_50_(s) < 15	20 ppm	<±1.0%
NO_2_ module			T_50_(s) < 10	5 ppm	<±0.5%
Particulate matter module	Humidity-corrected laser-scattering particle sensors	Module PMSX-003, Plantower Co., Ltd., Beijing	≤8 (s)	0.3~10 μm	PM_2.5_: ±10%@100~1000 μg m^−3^ ±10 μg m^−3^@0~100 μg m^−3^
Digital humidity sensor SHT7x (RH/T)	A capacitive polymer sensor and a precision thermistor sensor	SHT-75, Sensirion, Staefa, Switzerland	8 (s)	RH: 0–100% T: −40 to + 125 °C	Humidity accuracy%RH: ±1.8 Temperature °C: ±0.3
GPS module	GPS + GLONASS	-	-	-	-
Data transmission module	GSM	-	-	-	-

**Table 2:** NMSE and R for pollutants CO, NO_2_ and PM_2.5_.

	NMSE	R
Equation	$\frac{\underline{{\left(C_{o}-C_{p}\right)}^{2}}}{\underline{C_{o}\;}\underline{C_{p}}}$	$\frac{\underline{\left(C_{o}-\underline{C_{o}}\right)\left(C_{p}-\underline{C_{p}}\right)}}{\sigma C_{p}C_{o}}$
CO	0.016	0.996
NO_2_	0.007	0.953
PM_2.5_	0.021	0.948
Recommended criteria	<0.5	>0.8
Best agreement	0	1

**Table 3:** Average monthly coverage for each road type, average daily count, speed (km/h), total length (km), and number of segments.

Road Type	Average Monthly Coverage	Average Daily Count	Speed (km h^−1^)	Total Length (km)	Number of Segments
Trunk roads	95.1%	9.4	46.0 ± 16.0	788.8	6835
Motorways	80.4%	1.6	64.6 ± 20.2	1681.0	12,800
Primary roads	87.9%	8.7	39.8 ± 16.7	2399.1	15,975
Secondary roads	78.4%	9.6	33.9 ± 15.1	2563.0	15,508
Overall	84.1%	7.3	45.1 ± 20.9	7431.9	51,118

## Data Availability

The data may be obtained on request from the corresponding author.

## Supplementary Materials

The following supporting information can be downloaded at: https://www.mdpi.com/article/10.3390/s22166005/s1, Figure S1: Hourly average concentration, 5th percentile concentration and all AQMS hourly average Shanghai concentrations for CO (ppb), NO_2_ (ppb) and PM_2.5_ (μg m^−3^) in Shanghai in January 2020; Figure S2: Hourly average concentration, 5th percentile concentration and all AQMS hourly average Shanghai concentrations for CO (ppb), NO_2_ (ppb) and PM_2.5_ (μg m^−3^) in Shanghai in July 2020; Table S1: Real-time data table for time, device ID, GPS location (latitude and longitude), speed (m s^−1^), CO (ppb), NO_2_ (ppb), and PM_2.5_ (μg m^−3^) concentrations, temperature (°C), and relative humidity (%), the device voltage (V), the device status and GPS coordinates of Baidu map of each device in 5-s resolution; Table S2: Hourly average variation for CO (ppb), NO_2_ (ppb) and PM_2.5_ (μg m^−3^) in Shanghai from January 2020 to December 2020 on each type of roads.

## References

[1] Wróbel A., Rokita E., Maenhaut W. (2000). Transport of Traffic-Related Aerosols in Urban Areas. Sci. Total Environ..

[2] Satterthwaite D. (2009). The Implications of Population Growth and Urbanization for Climate Change. Environ. Urban..

[3] Lu Y., Wang Y., Zuo J., Jiang H., Huang D., Rameezdeen R. (2018). Characteristics of Public Concern on Haze in China and Its Relationship with Air Quality in Urban Areas. Sci. Total Environ..

[4] Power A.L., Tennant R.K., Jones R.T., Tang Y., Du J., Worsley A.T., Love J. (2018). Monitoring Impacts of Urbanisation and Industrialisation on Air Quality in the Anthropocene Using Urban Pond Sediments. Front. Earth Sci..

[5] Han X., Naeher L.P. (2006). A Review of Traffic-Related Air Pollution Exposure Assessment Studies in the Developing World. Environ. Int..

[6] Hubbell B.J., Kaufman A., Rivers L., Schulte K., Hagler G., Clougherty J., Cascio W., Costa D. (2018). Understanding Social and Behavioral Drivers and Impacts of Air Quality Sensor Use. Sci. Total Environ..

[7] Qiu G., Song R., He S. (2019). The Aggravation of Urban Air Quality Deterioration Due to Urbanization, Transportation and Economic Development—Panel Models with Marginal Effect Analyses across China. Sci. Total Environ..

[8] Urbanization United Nations Population Fund. https://www.unfpa.org/urbanization.

[9] Ambient(Outdoor) Air Pollution. https://www.who.int/news-room/fact-sheets/detail/ambient-(outdoor)-air-quality-and-health.

[10] Lim S.S., Vos T., Flaxman A.D., Danaei G., Shibuya K., Adair-Rohani H., AlMazroa M.A., Amann M., Anderson H.R., Andrews K.G. (2012). A Comparative Risk Assessment of Burden of Disease and Injury Attributable to 67 Risk Factors and Risk Factor Clusters in 21 Regions, 1990–2010: A Systematic Analysis for the Global Burden of Disease Study 2010. Lancet.

[11] Lin B., Zhu J. (2018). Changes in Urban Air Quality during Urbanization in China. J. Clean. Prod..

[12] Shao W., Zhang H., Zhou H. (2017). Fine Particle Sensor Based on Multi-Angle Light Scattering and Data Fusion. Sensors.

[13] Wu H., Gai Z., Guo Y., Li Y., Hao Y., Lu Z.-N. (2020). Does Environmental Pollution Inhibit Urbanization in China? A New Perspective through Residents’ Medical and Health Costs. Environ. Res..

[14] Molina L.T. (2021). Introductory Lecture: Air Quality in Megacities. Faraday Discuss..

[15] Zhang Y., Ye X., Wang S., He X., Dong L., Zhang N., Wang H., Wang Z., Ma Y., Wang L. (2021). Large-Eddy Simulation of Traffic-Related Air Pollution at a Very High Resolution in a Mega-City: Evaluation against Mobile Sensors and Insights for Influencing Factors. Atmos. Chem. Phys..

[16] Matz C.J., Egyed M., Hocking R., Seenundun S., Charman N., Edmonds N. (2019). Human Health Effects of Traffic-Related Air Pollution (TRAP): A Scoping Review Protocol. Syst. Rev..

[17] Wong P.P.Y., Lai P.-C., Allen R., Cheng W., Lee M., Tsui A., Tang R., Thach T.-Q., Tian L., Brauer M. (2019). Vertical Monitoring of Traffic-Related Air Pollution (TRAP) in Urban Street Canyons of Hong Kong. Sci. Total Environ..

[18] Zhu M., Zhang Z., Zhu B., Kong R., Zhang F., Tian J., Jiang T. (2020). Population and Economic Projections in the Yangtze River Basin Based on Shared Socioeconomic Pathways. Sustainability.

[19] Zhu W., Wang M., Zhang B. (2019). The Effects of Urbanization on PM2.5 Concentrations in China’s Yangtze River Economic Belt: New Evidence from Spatial Econometric Analysis. J. Clean. Prod..

[20] Hu X., Chen N., Wu N., Yin B. (2021). The Potential Impacts of Electric Vehicles on Urban Air Quality in Shanghai City. Sustainability.

[21] Air Pollution in China: Mapping of Concentrations and Sources|PLOS ONE. https://journals.plos.org/plosone/article?id=10.1371/journal.pone.0135749.

[22] Shi X., Zhao C., Jiang J.H., Wang C., Yang X., Yung Y.L. (2018). Spatial Representativeness of PM2.5 Concentrations Obtained Using Observations From Network Stations. J. Geophys. Res. Atmos..

[23] Zhang J., Reid J.S., Alfaro-Contreras R., Xian P. (2017). Has China Been Exporting Less Particulate Air Pollution over the Past Decade?. Geophys. Res. Lett..

[24] Zou B., You J., Lin Y., Duan X., Zhao X., Fang X., Campen M.J., Li S. (2019). Air Pollution Intervention and Life-Saving Effect in China. Environ. Int..

[25] Snyder E.G., Watkins T.H., Solomon P.A., Thoma E.D., Williams R.W., Hagler G.S.W., Shelow D., Hindin D.A., Kilaru V.J., Preuss P.W. (2013). The Changing Paradigm of Air Pollution Monitoring. Environ. Sci. Technol..

[26] Padilla L.E., Ma G.Q., Peters D., Dupuy-Todd M., Forsyth E., Stidworthy A., Mills J., Bell S., Hayward I., Coppin G. (2022). New Methods to Derive Street-Scale Spatial Patterns of Air Pollution from Mobile Monitoring. Atmos. Environ..

[27] Van Poppel M., Peters J., Bleux N. (2013). Methodology for Setup and Data Processing of Mobile Air Quality Measurements to Assess the Spatial Variability of Concentrations in Urban Environments. Environ. Pollut..

[28] Westerdahl D., Wang X., Pan X., Zhang K.M. (2009). Characterization of On-Road Vehicle Emission Factors and Microenvironmental Air Quality in Beijing, China. Atmos. Environ..

[29] Wei P., Brimblecombe P., Yang F., Anand A., Xing Y., Sun L., Sun Y., Chu M., Ning Z. (2021). Determination of Local Traffic Emission and Non-Local Background Source Contribution to on-Road Air Pollution Using Fixed-Route Mobile Air Sensor Network. Environ. Pollut..

[30] Apte J.S., Messier K.P., Gani S., Brauer M., Kirchstetter T.W., Lunden M.M., Marshall J.D., Portier C.J., Vermeulen R.C.H., Hamburg S.P. (2017). High-Resolution Air Pollution Mapping with Google Street View Cars: Exploiting Big Data. Environ. Sci. Technol..

[31] Yu Y.T., Xiang S., Li R., Zhang S., Zhang K.M., Si S., Wu X., Wu Y. (2022). Characterizing Spatial Variations of City-Wide Elevated PM10 and PM2.5 Concentrations Using Taxi-Based Mobile Monitoring. Sci. Total Environ..

[32] Haklay M., Weber P. (2008). OpenStreetMap: User-Generated Street Maps. IEEE Pervasive Comput..

[33] Zhang Y., Li X., Wang A., Bao T., Tian S. (2015). Density and Diversity of OpenStreetMap Road Networks in China. J. Urban Manag..

[34] Ballatore A., Zipf A., Fabrikant S.I., Raubal M., Bertolotto M., Davies C., Freundschuh S., Bell S. (2015). A Conceptual Quality Framework for Volunteered Geographic Information. Spatial Information Theory.

[35] Hagenauer J., Helbich M. (2012). Mining Urban Land-Use Patterns from Volunteered Geographic Information by Means of Genetic Algorithms and Artificial Neural Networks. Int. J. Geogr. Inf. Sci..

[36] Chu M., Brimblecombe P., Wei P., Liu C.-H., Du X., Sun Y., Yam Y.S., Ning Z. (2022). Kerbside NO_x_ and CO Concentrations and Emission Factors of Vehicles on a Busy Road. Atmos. Environ..

[37] Zong H., Brimblecombe P., Sun L., Wei P., Ho K.-F., Zhang Q., Cai J., Kan H., Chu M., Che W. (2021). Reducing the Influence of Environmental Factors on Performance of a Diffusion-Based Personal Exposure Kit. Sensors.

[38] Wei P., Ning Z., Ye S., Sun L., Yang F., Wong K., Westerdahl D., Louie P. (2018). Impact Analysis of Temperature and Humidity Conditions on Electrochemical Sensor Response in Ambient Air Quality Monitoring. Sensors.

[39] High Density Ozone Monitoring Using Gas Sensitive Semi-Conductor Sensors in the Lower Fraser Valley, British Columbia|Environmental Science & Technology. https://pubs.acs.org/doi/10.1021/es404610t.

[40] Cross E.S., Williams L.R., Lewis D.K., Magoon G.R., Onasch T.B., Kaminsky M.L., Worsnop D.R., Jayne J.T. (2017). Use of Electrochemical Sensors for Measurement of Air Pollution: Correcting Interference Response and Validating Measurements. Atmos. Meas. Tech..

[41] Zhang Z., Wang J., Hart J.E., Laden F., Zhao C., Li T., Zheng P., Li D., Ye Z., Chen K. (2018). National Scale Spatiotemporal Land-Use Regression Model for PM2.5, PM10 and NO2 Concentration in China. Atmos. Environ..

[42] Popoola O.A.M., Stewart G.B., Mead M.I., Jones R.L. (2016). Development of a Baseline-Temperature Correction Methodology for Electrochemical Sensors and Its Implications for Long-Term Stability. Atmos. Environ..

[43] Taylor J.R., Loescher H.L. (2013). Automated Quality Control Methods for Sensor Data: A Novel Observatory Approach. Biogeosciences.

[44] Van den Bossche J., Peters J., Verwaeren J., Botteldooren D., Theunis J., De Baets B. (2015). Mobile Monitoring for Mapping Spatial Variation in Urban Air Quality: Development and Validation of a Methodology Based on an Extensive Dataset. Atmos. Environ..

[45] Han S., Zhang Y., Wu J., Zhang X., Tian Y., Wang Y., Ding J., Yan W., Bi X., Shi G. (2015). Evaluation of Regional Background Particulate Matter Concentration Based on Vertical Distribution Characteristics. Atmos. Chem. Phys..

[46] Wu Y., Wang Y., Wang L., Song G., Gao J., Yu L. (2020). Application of a Taxi-Based Mobile Atmospheric Monitoring System in Cangzhou, China. Transp. Res. Part D Transp. Environ..

[47] Kumar A., Luo J., Bennett G.F. (1993). Statistical Evaluation of Lower Flammability Distance (LFD) Using Four Hazardous Release Models. Process Saf. Progress.

[48] Bukowiecki N., Dommen J., Prévôt A.S.H., Richter R., Weingartner E., Baltensperger U. (2002). A Mobile Pollutant Measurement Laboratory—Measuring Gas Phase and Aerosol Ambient Concentrations with High Spatial and Temporal Resolution. Atmos. Environ..

[49] Brantley H.L., Hagler G.S.W., Kimbrough E.S., Williams R.W., Mukerjee S., Neas L.M. (2014). Mobile Air Monitoring Data-Processing Strategies and Effects on Spatial Air Pollution Trends. Atmos. Meas. Tech..

[50] (2014). Brake and Tire Wear Emissions from On-Road Vehicles in MOVES2014.

[51] Sanders P.G., Xu N., Dalka T.M., Maricq M.M. (2003). Airborne Brake Wear Debris:  Size Distributions, Composition, and a Comparison of Dynamometer and Vehicle Tests. Environ. Sci. Technol..

[52] Garg B.D., Cadle S.H., Mulawa P.A., Groblicki P.J., Laroo C., Parr G.A. (2000). Brake Wear Particulate Matter Emissions. Environ. Sci. Technol..

[53] Hu X., Zhang Y., Ding Z., Wang T., Lian H., Sun Y., Wu J. (2012). Bioaccessibility and Health Risk of Arsenic and Heavy Metals (Cd, Co, Cr, Cu, Ni, Pb, Zn and Mn) in TSP and PM2.5 in Nanjing, China. Atmos. Environ..

[54] Yan Y., Li Y., Sun M., Wu Z. (2019). Primary Pollutants and Air Quality Analysis for Urban Air in China: Evidence from Shanghai. Sustainability.

[55] Sokhi R.S., Singh V., Querol X., Finardi S., Targino A.C., de Fatima Andrade M., Pavlovic R., Garland R.M., Massagué J., Kong S. (2021). A Global Observational Analysis to Understand Changes in Air Quality during Exceptionally Low Anthropogenic Emission Conditions. Environ. Int..

[56] Cooper M.J., Martin R.V., Hammer M.S., Levelt P.F., Veefkind P., Lamsal L.N., Krotkov N.A., Brook J.R., McLinden C.A. (2022). Global Fine-Scale Changes in Ambient NO2 during COVID-19 Lockdowns. Nature.

[57] Wu C., Wang H., Cai W., He H., Ni A., Peng Z. (2021). Impact of the COVID-19 Lockdown on Roadside Traffic-Related Air Pollution in Shanghai, China. Build. Environ..

[58] Liu C., Hua C., Kang Z. (2017). Characteristics of Air Pollution and Its Resources During Winter and Summer Seasons of 2014 and 2015 in Shanghai. Meteorol. Mon..

[59] Xu J., Chang L., Ma J., Mao Z., Chen L., Cao Y. (2016). Objective Synoptic Weather Classification on PM2.5 Pollution during Autumn and Winter Seasons in Shanghai. Acta Sci. Circumst..

[60] Chen K., Wang M., Huang C., Kinney P.L., Anastas P.T. (2020). Air Pollution Reduction and Mortality Benefit during the COVID-19 Outbreak in China. Lancet Planet. Health.

