# Supplementary material for: High Resolution On-Road Air Pollution Using a Large Taxi-Based Mobile Sensor Network
Source: Sensors (Basel). 2022 Aug 11;22(16):6005. doi: 10.3390/s22166005 (PMC9416088; doi:10.3390/s22166005)
Supplement: Supplementary file 1 [file sensors-22-06005-s001.zip › sensors-1800548-supplementary.pdf]

Supplementary Information

PostTime	MN	LON	LAT	SPEED	CO	NO_2	$PM_{2.5}$	TEMPERATURE	HUMIDITY	VOLTAGE	Flag	LON_BAIDU	LAT_BAIDU
2020/01/01 00:00:02	TX004	121.380449	31.216119	32	17	52	35	5.8	51	11.3	N	121.3916911	31.21991913
2020/01/01 00:00:02	TX007	121.375828	31.213605	38	20	34	94	4.2	55	11.7	N	121.3870659	31.21738619
2020/01/01 00:00:02	TX101	121.25672	31.378435	31	34	60	64	4.7	54	11.8	N	121.2677577	31.38210837
2020/01/01 00:00:02	TX102	121.321421	31.188136	13	19	34	84	5	53	11.6	N	121.3323433	31.19251894
2020/01/01 00:00:02	TX106	121.615954	31.265838	1	8	18	47	6.6	47	10.2	N	121.6267526	31.26929257
2020/01/01 00:00:02	TX108	121.604279	31.314497	1	13	22	55	6.8	50	10.5	N	121.6150568	31.31802836
2020/01/01 00:00:02	TX117	121.44619	31.291809	91	17	32	53	7	45	11.2	N	121.457197	31.29626059
2020/01/01 00:00:02	TX121	121.464042	31.226581	1	22	46	66	7.4	45	11.6	N	121.4751066	31.23069376

Table S1: Real-time data table for time, device ID, GPS location (latitude and longitude), speed (m s^{-1}), CO (ppb), NO_2 (ppb), and $PM_{2.5}$ (μg m^{-3}) concentrations, temperature (°C), and relative humidity (%), the device voltage (V), the device status and GPS coordinates of Baidu map of each device in 5-second resolution.

Roadway types	CO (ppb)		NO_2 (ppb)		$PM_{2.5}$ (μg/m^3)	
	Average ± Sd	CV%	Average ± Sd	CV%	Average ± Sd	CV%
Trunk	986.69±274.67	27.84%	91.68±16.16	17.63%	56.10±7.74	13.80%
Motorways	780.41±233.83	29.96%	113.10±27.98	24.74%	61.19±14.43	23.58%
Primary roads	880.24±228.69	25.98%	93.95±30.53	32.50%	58.96±12.65	21.46%
Secondary roads	826.32±269.96	32.67%	82.17±23.38	28.45%	57.71±17.73	30.72%
Overall	854.13±257.61	30.16%	94.97±28.65	30.17%	58.75±14.37	24.46%

Table S2: Hourly average variation for CO (ppb), NO_2 (ppb) and $PM_{2.5}$ (μg m^{-3}) in Shanghai from January 2020 to December 2020 on each type of roads.

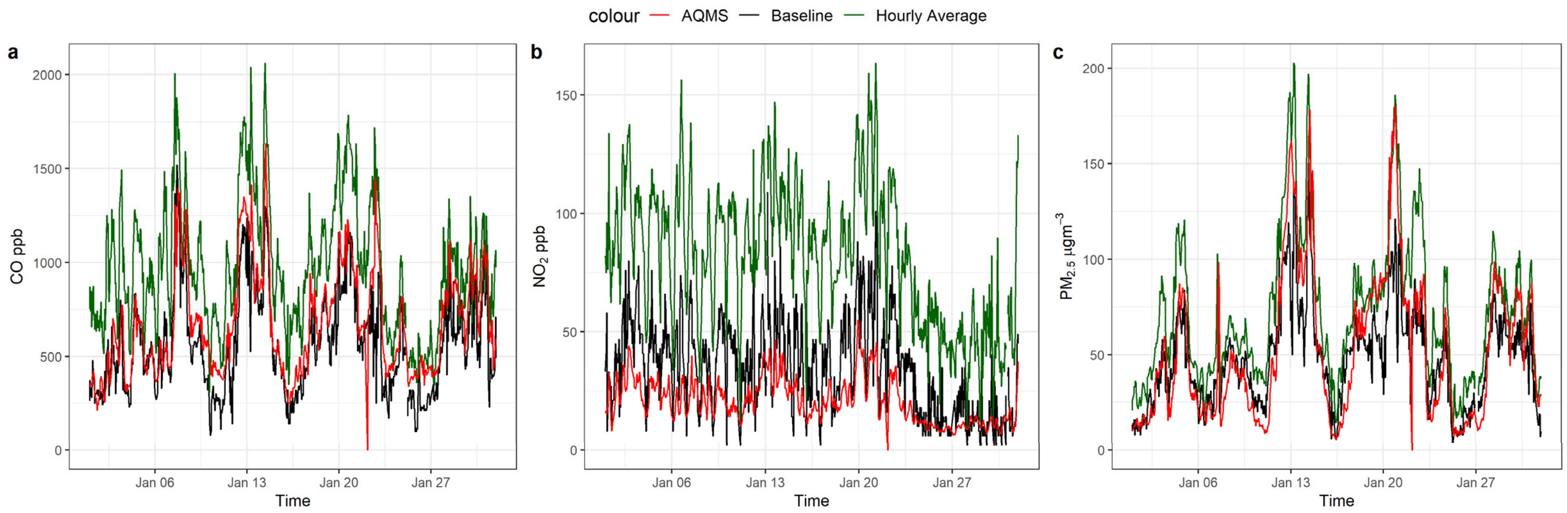

Figure S1: Hourly average concentration, 5th percentile concentration and all AQMS hourly average Shanghai concentrations for CO (ppb), NO_2 (ppb) and $PM_{2.5}$ ($\mu g\ m^{-3}$) in Shanghai in January 2020.

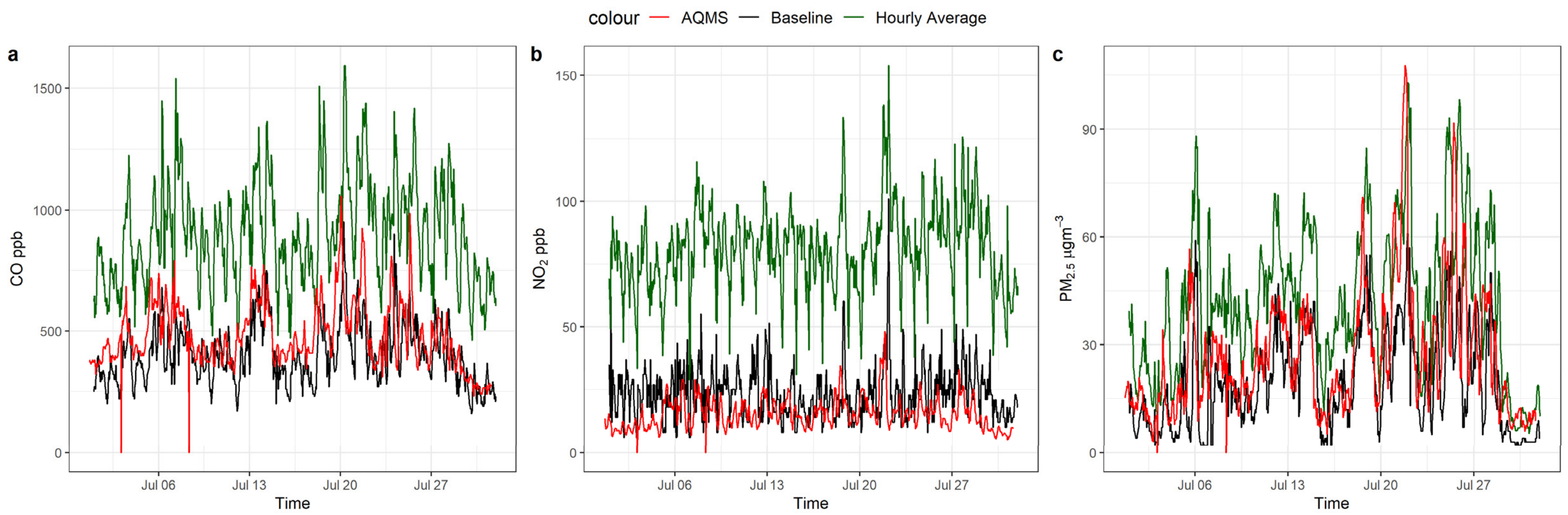

Figure S2: Hourly average concentration, 5th percentile concentration and all AQMS hourly average Shanghai concentrations for CO (ppb), NO_2 (ppb) and $PM_{2.5}$ (µg m^{-3}) in Shanghai in July 2020.
